# Superior vena cava perforation during atrial lead implantation in a patient with repaired tetralogy of Fallot: a case report

**DOI:** 10.1093/ehjcr/ytag680

**Published:** 2026-09-10

**Authors:** Ibrahim Antoun, Abdul Hanan Hamid, Mohammad El-Din, Kassem Safwan

**Affiliations:** Department of Cardiology, Kettering General Hospital, Kettering NN16 8UZ, UK; Division of Cardiovascular Sciences, University of Lciester, Leicester LE3 9QP, UK; Department of Cardiology, Kettering General Hospital, Kettering NN16 8UZ, UK; Department of Cardiology, Kettering General Hospital, Kettering NN16 8UZ, UK; Department of Cardiology, Kettering General Hospital, Kettering NN16 8UZ, UK; Department of Cardiology, Glenfield Hospital, Leicester LE3 9QP, UK

**Keywords:** Pacemaker lead, Complication, Tetralogy of Fallot, Adult congenital heart disease, Case report

## Abstract

**Background:**

Superior vena cava (SVC) injury during pacemaker implantation is rare, with most cases reported during lead extraction rather than placement. In adults with congenital heart disease (ACHD), unexpected resistance during lead advancement requires immediate reassessment because technical or device-related factors can cause venous injury even when pre-procedural imaging does not show hostile venous anatomy. Evidence on the management of venous injury during implantation is limited.

**Case presentation:**

A 62-year-old woman with repaired tetralogy of fallot (TOF) presented with recurrent syncope. An implantable loop recorder documented a 10-s sinus pause. She underwent elective dual-chamber pacemaker implantation. Left subclavian venous access was obtained, and the right ventricular lead was positioned without difficulty. During advancement of the atrial lead, resistance was encountered at the subclavian–SVC junction, and the lead was withdrawn. Intraprocedural venography demonstrated focal contrast pooling at the SVC–azygos junction with mediastinal extravasation, concerning for SVC dissection or contained perforation. The patient remained haemodynamically stable. Transthoracic echocardiography showed no pericardial effusion. Non-contrast computed tomography of the chest confirmed mediastinal contrast adjacent to a patent SVC without obstruction or thrombosis. Conservative management with close observation was undertaken. Repeat CT imaging after 24 h demonstrated complete resolution. Inspection of the atrial lead revealed unintended extension of the active-fixation screw, suggesting a mechanical mechanism for venous injury.

**Discussion:**

This case highlights a rare but important complication of pacemaker implantation in ACHD. The normal pre-procedural venous assessment, uncomplicated right ventricular lead placement, and unintended extension of the atrial lead fixation screw support a device-related mechanical mechanism rather than anatomical difficulty. It emphasizes immediate cessation of advancement when resistance occurs, lead inspection with confirmation of screw retraction, early venography, and conservative management in haemodynamically stable patients with a patent SVC.

Learning points

- SVC dissection or contained perforation should be suspected when resistance during lead advancement is followed by contrast pooling or mediastinal extravasation.
- Conservative management can be appropriate in haemodynamically stable patients without pericardial effusion, haemothorax, venous obstruction, or falling haemoglobin.
- Active fixation leads should be inspected before insertion to confirm complete screw retraction, and unexpected resistance should prompt withdrawal, lead inspection, and venography before further manipulation.

## Background

Pacemaker implantation is a common and generally safe procedure. However, venous complications, such as thrombosis, stenosis, dissection, and perforation, although rare, may occur.^1^ Adults with congenital heart disease (ACHD) increasingly require cardiac implantable electronic devices for indications, including sinus node dysfunction and atrioventricular conduction disease, and often require individualized procedural planning.^2,3^

Although repaired TOF can be associated with procedural complexity in some patients,^4,5^ the available pre-procedural cardiac magnetic resonance (CMR) in this patient did not demonstrate hostile venous anatomy, and the right ventricular lead advanced without difficulty. These observations made a fixed anatomical cause of the subsequent resistance less likely. Superior vena cava (SVC) injury is most frequently reported during transvenous lead extraction, where major tears carry significant morbidity and mortality.^6^ Reports of SVC injury during lead implantation are extremely limited.

This case describes a suspected SVC dissection or contained perforation occurring during atrial lead manipulation in a repaired TOF patient, successfully managed with a conservative strategy.

## Summary figure

**Figure:**
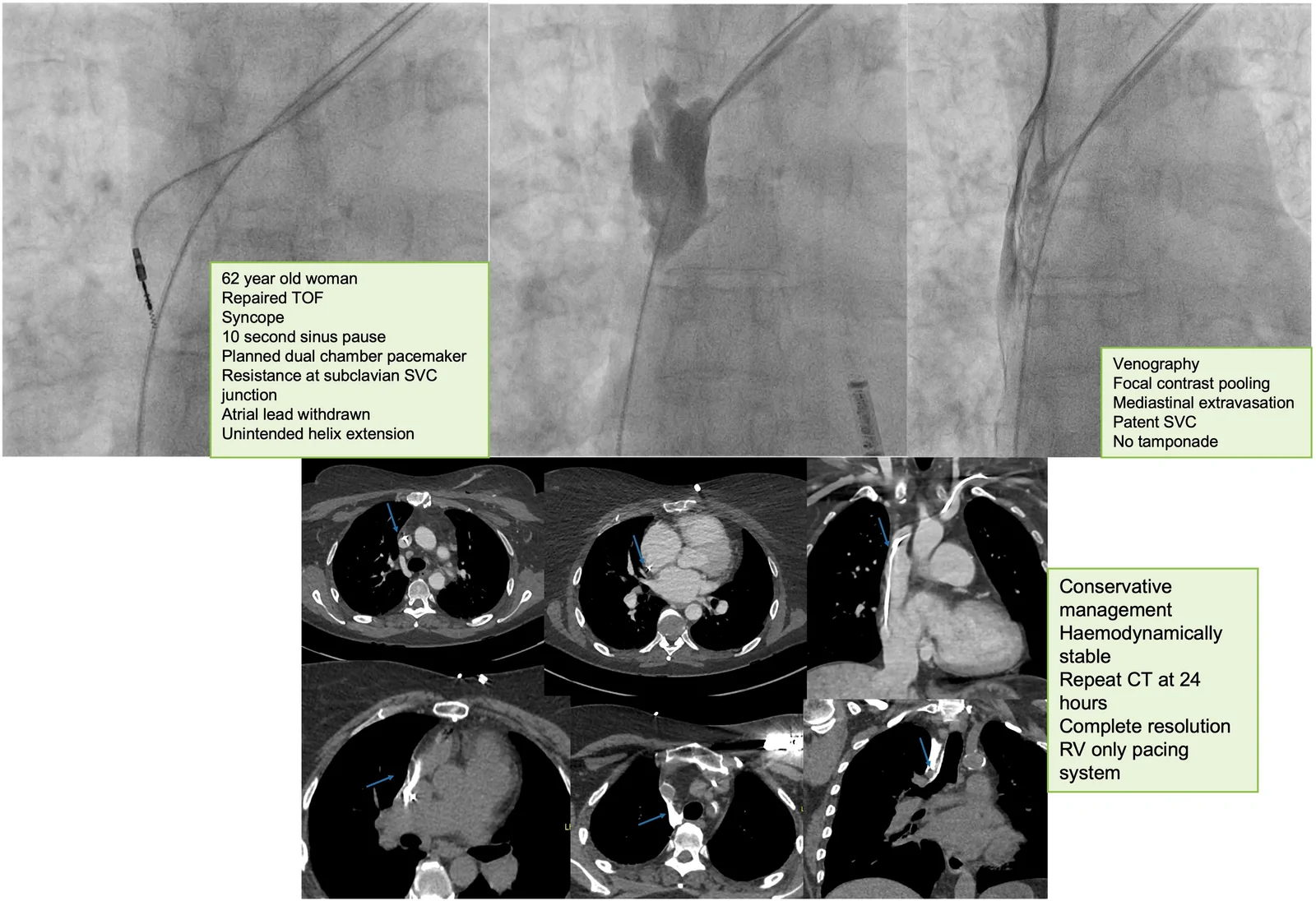


## Case presentation

A 62-year-old woman with repaired Tetralogy of Fallot in childhood attended routine adult congenital heart disease follow-up. Cardiac magnetic resonance imaging demonstrated severe pulmonary regurgitation, a non-dilated right ventricle with preserved systolic function, and a non-dilated left ventricle with mild systolic dysfunction. She reported recurrent episodes of syncope. An implantable loop recorder documented a 10-s sinus pause, consistent with significant sinus node dysfunction.

**Figure 1:**
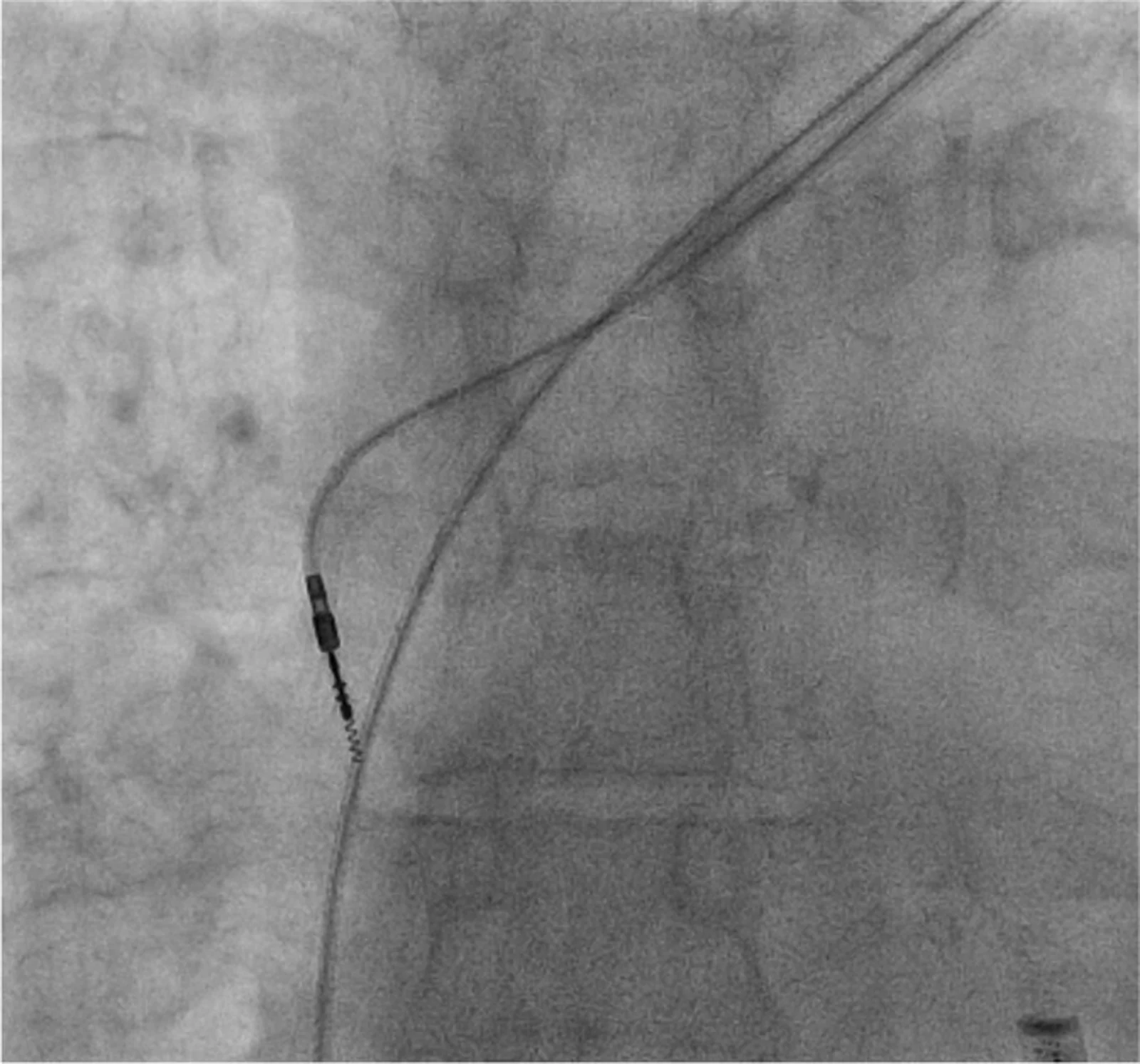
Unintended extension of active-fixation screw on atrial pacing lead. Fluoroscopic image of the right atrial pacing lead demonstrating protrusion of the active-fixation helix despite no intentional deployment, providing a plausible mechanical explanation for superior vena cava wall injury encountered during attempted lead advancement.

She was scheduled for elective dual-chamber pacemaker implantation. Pre-procedural CMR was available and did not demonstrate high-risk venous features to suggest hostile access anatomy. Therefore, initial venography was not performed. Left subclavian venous access was obtained, and the right ventricular lead was advanced and positioned without difficulty. During advancement of the right atrial lead, resistance was encountered at the level of the subclavian–SVC junction (*Figure 1*). The lead was withdrawn immediately.

**Figure 2:**
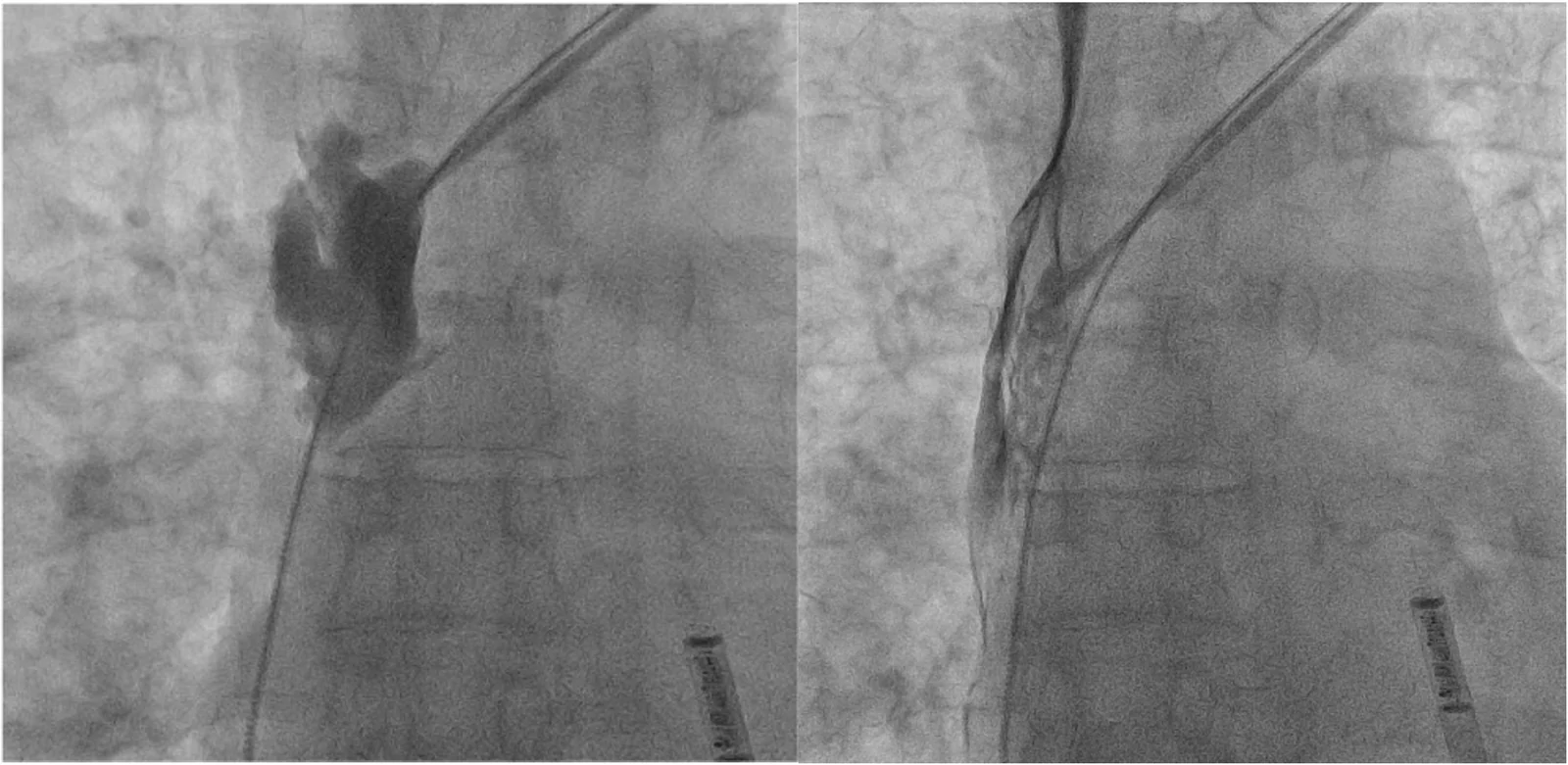
Contrast venography showing focal superior vena cava contrast extravasation. Intraprocedural contrast injection at the subclavian–superior vena cava junction demonstrates localized contrast pooling with extravasation into the mediastinum, consistent with superior vena cava dissection or contained venous perforation encountered during attempted atrial lead advancement.

Contrast venography was performed and demonstrated focal contrast pooling within the SVC at the level of the azygos vein entry, with mediastinal contrast extravasation, raising concern for SVC dissection or contained perforation (*Figure 2*). The patient remained haemodynamically stable and asymptomatic, with no chest pain, dyspnoea, or desaturation.

The right ventricular lead was connected to the generator, and the device pocket was closed without further atrial lead manipulation. Immediate transthoracic echocardiography showed no pericardial effusion or features of tamponade. Non-contrast computed tomography of the chest demonstrated mediastinal contrast adjacent to the SVC without luminal obstruction, thrombosis, or large haematoma, supporting a diagnosis of SVC dissection or contained venous perforation rather than free rupture. Differential considerations included venous intimal injury without full-thickness perforation or transient contrast extravasation related to focal venous injury, while significant venous stenosis was considered less likely given preserved vessel patency.

**Figure 3:**
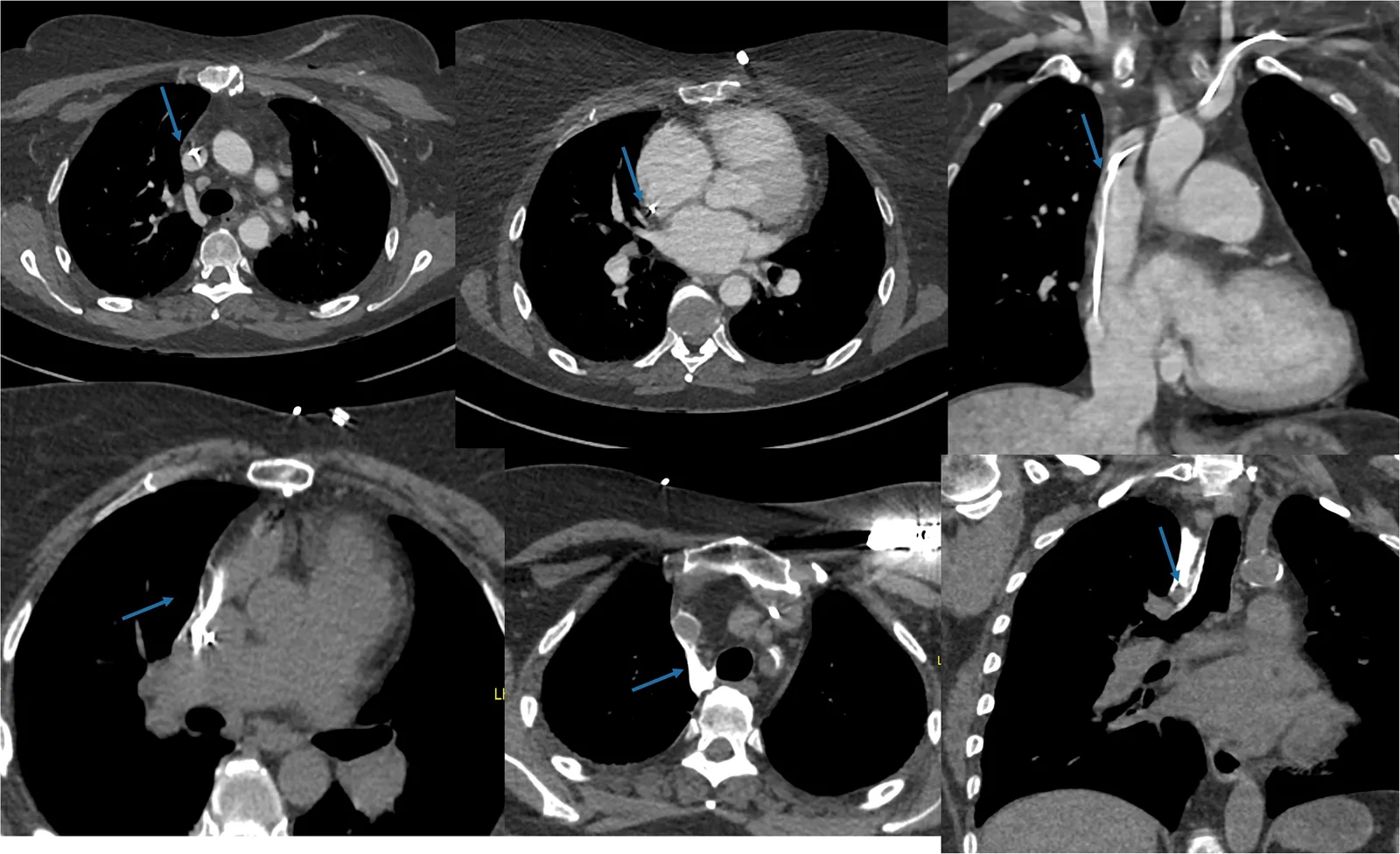
Computed tomography of superior vena cava injury. Axial and coronal non-contrast CT images of the chest demonstrating contrast tracking and mediastinal extravasation adjacent to the superior vena cava at the level of the azygos vein junction (blue arrows), consistent with superior vena cava dissection or contained perforation. The superior vena cava lumen remains patent with no evidence of thrombosis or external compression.

Given the absence of haemodynamic compromise, preserved SVC flow, and lack of ongoing bleeding, a conservative management strategy was adopted with close clinical observation. Serial haemodynamic assessment and haemoglobin monitoring were performed during observation and showed no evidence of ongoing bleeding. CT imaging performed 24 h later demonstrated complete resolution of mediastinal contrast and an unobstructed SVC. CT imaging performed 24 h later demonstrated complete resolution of mediastinal contrast and an unobstructed SVC (*Figure 3*).

Inspection of the removed atrial lead revealed unintended extension of the active-fixation screw despite no deliberate deployment, suggesting a mechanical mechanism for venous wall injury. This device’s abnormality was reported to the manufacturer.

The patient remained clinically stable throughout admission. The original indication for dual-chamber pacing remained symptomatic sinus node dysfunction with a documented 10-second sinus pause. However, given the acute venous injury and adequate ventricular backup pacing, further atrial lead manipulation was deferred. She was discharged with a functioning right ventricular-only pacing system and continues to do well at subsequent ACHD and device clinic follow-up. A renewed attempt at atrial lead implantation may be considered after complete healing if clinically required, with pre-procedural venous imaging or venography and explicit confirmation of active-fixation screw retraction before lead advancement.

## Discussion

The sequence of events supports a device-related mechanical injury rather than an anatomical complication. Pre-procedural CMR did not show hostile venous features, the right ventricular lead traversed the access route without difficulty, and resistance occurred only during atrial lead advancement. Inspection of the removed atrial lead then demonstrated unintended extension of the active-fixation screw, creating a plausible sharp leading point capable of injuring the venous wall. The repaired TOF therefore provides important clinical context but was not demonstrated to be the cause of the SVC injury. The procedure was elective, without emergency time pressure or deliberate deviation from standard implantation practice. This event highlights how a rare device-related abnormality may go unnoticed unless the fixation mechanism is systematically checked before insertion.^7^ Venous complications related to pacing leads more commonly include thrombosis, stenosis, or SVC syndrome, sometimes necessitating endovascular or surgical intervention.^8^

SVC injury is most frequently reported during transvenous lead extraction, where major tears are associated with significant mortality.^6^ In contrast, reports of SVC injury during lead implantation are extremely rare. Iatrogenic venous injury from intravascular devices has occasionally been described, with conservative management proving successful in haemodynamically stable patients without ongoing bleeding or venous obstruction.^9^

The unintended extension of the active-fixation screw provides the most plausible mechanism of venous injury in this case. A partially extended helix may act as a penetrating element and disrupt the venous intima during lead advancement. Importantly, unexpected resistance should not be attributed to presumed congenital anatomical complexity without confirmation, because this may delay recognition of a technical or device-related problem. When resistance is encountered, advancement should stop immediately. The lead should be withdrawn and inspected, including explicit confirmation that the active-fixation helix is fully retracted, and venography should be performed before any further manipulation. Following this event, our procedural workflow was modified to make pre-insertion confirmation of complete helix retraction a mandatory safety step for active-fixation leads.^10^

Pre-procedural venous imaging remains useful when specific risk features are suspected, but it was not explanatory in this case. The available CMR showed no high-risk venous features, and the uncomplicated passage of the right ventricular lead further reduced suspicion of a fixed anatomical obstacle. Accordingly, the value of pre-procedural imaging here is to document the absence of an anticipated access problem rather than to explain the complication.^11^

The case also underscores the diagnostic value of intraprocedural venography when unexpected resistance is encountered. Early contrast injection can distinguish mechanical obstruction from vascular injury and prompt immediate procedural modification before further lead manipulation. Once venous injury was identified, the patient's haemodynamic stability, preserved SVC patency, absence of ongoing bleeding, and lack of pericardial effusion supported conservative management with close observation, serial haemoglobin assessment, and repeat CT imaging. Complete radiological resolution at 24 h supported this strategy.^9^ Targeted venography or careful review of recent cross-sectional imaging remains reasonable before implantation when there is known or suspected venous stenosis, prior central venous instrumentation, collateralization, or other specific concern about access.^12^ Future workflow tools, including AI-assisted imaging review and device-check protocols, may support systematic identification of anatomical or technical hazards before implantation.^13,14^

Finally, this report highlights an under-recognized device-related factor. Manufacturing or packaging-related partial extension of active-fixation screws, although likely rare, may represent a latent hazard. Systematic pre-insertion verification of complete screw retraction should be treated as a mandatory safety step for all active-fixation leads, regardless of the patient's underlying anatomy.^15^

## Lead author biography


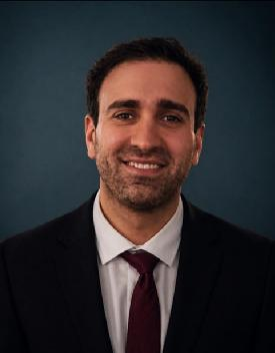


Ibrahim Antoun graduated from the University of Aleppo with an MD degree in 2016. He is a UK-licensed physician and a member of the Royal College of Physicians since 2021. Completed his PhD in Electrophysiology at the University of Leicester in 2023. Currently works as a Cardiology trainee in the East Midlands deanery and as an honorary research fellow at the University of Leicester. Obtained Fellowship of the European Society of Cardiology in 2025. Has over 100 peer-reviewed articles in the field of cardiology, arrhythmia care and non-communicable diseases in low-to middle-income countries. He is a certified cardiac devices specialist (CCDS).

## Data Availability

Data regarding this case report is available on request from the corresponding author.
